# Unity gives strength: combining Bertaut’s and Belov’s concepts and the formalism of aperiodic crystals to solve magnetic structures of unprecedented complexity

**DOI:** 10.1107/S2052252524010182

**Published:** 2024-10-29

**Authors:** Václav Petříček, Margarida Sousa Henriques

**Affiliations:** a Structure Analysis Institute of Physics of the Czech Academy of Sciences Na Slovance 1999/2 Prague 182 00 Czechia

**Keywords:** structure analysis, magnetic structures, modulated structures, magnetic phase transitions

## Abstract

We draw attention to the exceptional work of Geers *et al.*[(2024). *IUCrJ*, **11**, 910–920] on the analysis of magnetic phases, in which challenging magnetic structures are determined by a combination of modern computational methods and a connection between nuclear modulation and the ordering of magnetic moments is shown.

Crystal structure analysis from diffraction data is a method that has been around for over a hundred years. Collecting data for and then solving simple structures takes only a few hours and is a routine procedure that no longer surprises anyone. Yet structural analysis continues to fascinate us by opening up new avenues and possibilities. The paper by Geers *et al.* (2024 ▸) in this issue of **IUCrJ** is an excellent example of this, as it illustrates the tremendous progress that has been made in the last decade in solving magnetic structures from neutron diffraction data.

The article by Geers *et al.* is one of very few reports on modulated molecular frameworks. It summarizes the results of determining the modulated magnetic structure of the metal–organic coordination polymers [CH_3_NH_3_]Co_*x*_Ni_1−*x*_(HCOO)_3_, a series of compounds in which very unusual phase transitions have been observed. The authors describe how the magnetic ordering in the structures and magnetic phase transitions depend on the metal and its environment in the complex (Fig. 1 ▸). Furthermore, they show that it is possible to choose the pathway to the magnetic transition and tune the critical temperature and stability of the magnetic phase. It is remarkable how the magnetic state is reached via at least one modulated crystal phase. Strikingly, some phases show a combined nuclear and magnetic modulation at the lowest temperatures, indicating that a periodically deformed structure leads directly to a wave in the spin distribution. Therefore, the study is essential for understanding the interplay between the arrangement of atoms in the crystal and the magnetic moments.

The authors combine two seemingly different approaches to study the ordering of the magnetic moments in a crystal: representation analysis, based on irreducible representations of the initial paramagnetic phase as described by Bertaut (1968 ▸), and the description of magnetic ordering using magnetic groups as given by Belov *et al.* (1957 ▸). It has recently been shown that by combining these methods the arrangement of the atoms and magnetic moments in individual phases can be solved much more easily and to a better degree. The fundamental problem for determining the proper, rather than the apparent, symmetry is that neutron diffraction combines diffraction effects from the atomic nuclei and from the magnetic moments. These can fully overlap in cases where the arrangement of the magnetic moments does not lead to a violation of translational symmetry. The violation of translational symmetry by the arrangement of the magnetic moments is commensurate when the propagation vector is half of one of the fundamental vectors of the primitive cell, but incommensurate when the propagation vector has general components. Commensurate cases are covered by magnetic space-group type IV. However, for incommensurate structures, it is necessary to use the superspace formalism introduced by de Wolff *et al.* (1981 ▸), which was later extended to magnetic structures by Perez-Mato *et al.* (2012 ▸) as magnetic superspace groups (MSSGs). These allow the symmetry of the crystal to be understood as including positional, occupation and magnetic modulations.

Determining the MSSG from experimental data is not easy at all. In most cases, the diffraction image simulates a symmetry that is higher than the real point symmetry because magnetic phase transitions lead, in most cases, to the existence of a number of magnetic domains with the same fractional volume. Moreover, systematic extinction of diffraction is complicated by the fact that the structure factor of the magnetic structure is an axial vector, and therefore all its components must meet the extinction conditions. The combination of representation analysis with the MSSG formalism helps to solve the situation, as implemented in the highly sophisticated tool ISODISTORT (Stokes *et al.*, 2023 ▸; Campbell *et al.* 2006 ▸), which finds possible MSSGs based on knowledge of the initial structure and the propagation vector.

In the work of Geers *et al.*, the methods mentioned above are applied to the end members of the series of solid solutions, *i.e.* those containing only nickel or cobalt, measured at different temperatures. The differences in the behavior of these compounds are noteworthy, as shown in their Fig. 1 ▸. The first phase transformation has the same character for both crystals, in which an unmodulated structure transforms into a modulated one with the same superspace group and similar modulation vectors. A further decrease in temperature leads, in the case of the nickel compound, to a modulated structure where both the atomic position and magnetic moment of the nickel atom are modulated, while the cobalt compound undergoes two phase transitions: from the orthorhombic to a monoclinic group (twinned) and then to the magnetic space group *P*2_1_′/*n*′.

Apart from the compounds containing only nickel or cobalt, the authors also studied in detail the solid solutions that contained a mixture of both metals (*x* = 0.25, 0.5 and 0.75) and discovered a full landscape of modulated and magnetic phases (see Fig. 5 in their paper). Finally, we would like to draw the reader’s attention to the information provided in their supporting information, which serves as an excellent introduction to solving and refining magnetic structures.

In summary, the work of Geers *et al.* proves that the analysis of crystal structures by diffraction methods is still open to new challenges. It also hints at a possible role of modulated crystal structures as a controlling mechanism for spin degrees of freedom in these materials via selected crystal phase transitions.

## Figures and Tables

**Figure 1:**
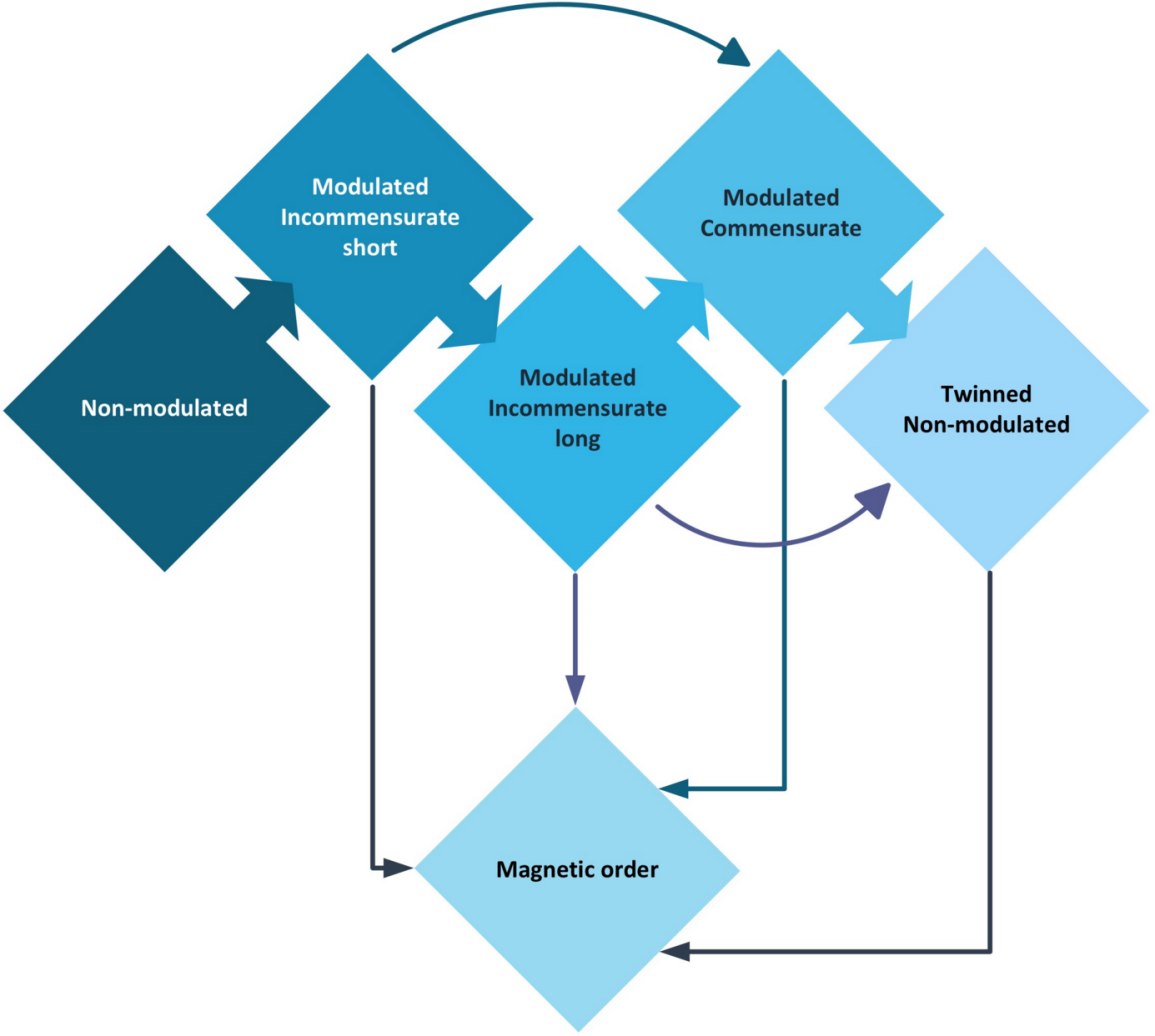
Pathways to reaching the magnetically ordered phase in the system studied by Geers *et al.* (2024) ▸. Possible crystal phase transition sequences are either direct (short arrows between the lozenges in the upper row) or indirect (curved arrows), depending on the substitution of Ni by Co. The magnetically ordered phase can be reached from any of the crystal phases linked to it by straight arrows.
